# Capsid Structure of dsRNA Fungal Viruses

**DOI:** 10.3390/v10090481

**Published:** 2018-09-07

**Authors:** Daniel Luque, Carlos P. Mata, Nobuhiro Suzuki, Said A. Ghabrial, José R. Castón

**Affiliations:** 1 Department of Structure of Macromolecules, Centro Nacional de Biotecnología (CNB-CSIC), Campus Cantoblanco, 28049 Madrid, Spain; dluque@isciii.es (D.L.); cpmata@mrc-lmb.cam.ac.uk (C.P.M.); 2 Centro Nacional de Microbiología/ISCIII, Majadahonda, 28220 Madrid, Spain; 3 Institute of Plant Science and Resources, Okayama University, Kurashiki 710-0046, Japan; nsuzuki@okayama-u.ac.jp; 4 Department of Plant Pathology, University of Kentucky, Lexington, KY 40546, USA; saghab00@uky.edu

**Keywords:** dsRNA virus, mycovirus, capsid protein, capsid structure, virus evolution, viral lineage, ScV-L-A, PcV, PsV-F, RnQV1

## Abstract

Most fungal, double-stranded (ds) RNA viruses lack an extracellular life cycle stage and are transmitted by cytoplasmic interchange. dsRNA mycovirus capsids are based on a 120-subunit T = 1 capsid, with a dimer as the asymmetric unit. These capsids, which remain structurally undisturbed throughout the viral cycle, nevertheless, are dynamic particles involved in the organization of the viral genome and the viral polymerase necessary for RNA synthesis. The atomic structure of the T = 1 capsids of four mycoviruses was resolved: the L-A virus of *Saccharomyces cerevisiae* (ScV-L-A), *Penicillium chrysogenum* virus (PcV), *Penicillium stoloniferum* virus F (PsV-F), and *Rosellinia necatrix* quadrivirus 1 (RnQV1). These capsids show structural variations of the same framework, with 60 asymmetric or symmetric homodimers for ScV-L-A and PsV-F, respectively, monomers with a duplicated similar domain for PcV, and heterodimers of two different proteins for RnQV1. Mycovirus capsid proteins (CP) share a conserved α-helical domain, although the latter may carry different peptides inserted at preferential hotspots. Insertions in the CP outer surface are likely associated with enzymatic activities. Within the capsid, fungal dsRNA viruses show a low degree of genome compaction compared to reoviruses, and contain one to two copies of the RNA-polymerase complex per virion.

## 1. Introduction

Double-stranded RNA (dsRNA) viruses infect a diversity of host organisms, from bacteria to unicellular and simple eukaryotes (fungi and protozoa), through to plants and animals. No archaea-infecting dsRNA viruses have yet been reported [1]. Although dsRNA viruses are a rather diverse group, they share general architectural principles and numerous functional features. The complexity of the capsid ranges from a single shell [2,3] to a multilayered and concentric capsid [4,5,6,7]. Whereas the outer shell has a protective role and is involved in cell entry, the innermost capsid (or inner core), which all these viruses possess, is devoted to the organization of the viral genome and viral polymerase. As a whole this specialized capsid consists of 120 protein subunits arranged in a T = 1 icosahedral shell, i.e., a capsid protein (CP) dimer is the asymmetric unit. This T = 1 capsid is also referred as a “T = 2 layer”—an exception to the quasi-equivalence theory proposed by Caspar and Klug [8].

The T = 1 capsids of dsRNA viruses are known to be critical for genome replication (minus-strand synthesis) and transcription (plus-strand synthesis), with the viral RNA-dependent RNA polymerase(s) (RdRp) frequently packaged as an integral component of the capsid. T = 1 capsids also function as molecular sieves, allowing the exit of single-stranded (ss) RNA transcripts for translation in the host cytoplasm, and the entrance of nucleotides for intra-capsid RNA synthesis. The pores are presumably small enough to exclude potentially degradative enzymes.

T = 1 capsids remain structurally undisturbed throughout the viral cycle [9], isolating dsRNA molecules and any replicative intermediates, thus preventing the triggering of dsRNA sensor-mediated antiviral host defense mechanisms, such as RNA silencing, interferon synthesis, and apoptosis [10,11,12].

The totiviruses ScV-L-A and UmV-P4, which infect the yeast *Saccharomyces cerevisiae* and the smut fungus *Ustilago maydis* respectively, were the first unambiguously described viruses with a T = 1 capsid formed by 12 decamers rather than 12 pentamers [13]. The conservation of this stoichiometry and architecture is probably related to the stringent requirements of capsid RNA metabolism-associated activity [14,15] as the capsid organizes the packaged genome and the replicative complex(es). RdRp is incorporated as a replicative complex at the pentameric vertex (as in rotavirus capsids [16,17]), as a fusion protein with the CP (as in the totivirus ScV-L-A [3]), or as a separate, non-fused protein (as in the victorivirus HvV190SV [18]).

The 120-subunit T = 1 capsids have been described for members of the *Reo*- [19,20,21,22,23] and *Picobirnaviridae* [24], which mostly infect higher eukaryotic organisms. They have also been described for members of the family *Cystoviridae*—bacteriophages that infect the prokaryote *Pseudomonas syringae* [25,26]. Members of the Toti- [27,28,29], Partiti- [30,31], Megabirna- [32], Chryso- [33,34,35], and Quadriviridae [36,37] families, which infect unicellular and simple eukaryotes, such as fungi, protozoa, but also some plants, also have these capsids. Members of the family Birnaviridae, which infect vertebrates, mollusks, insects, and rotifers, are exceptions, since they lack the T = 1 core of 60 CP dimers [38,39]. Rather, these have a single T = 13 shell that encapsidates a polyploid dsRNA genome organized as ribonucleoprotein complexes [40,41].

**Figure 1:**
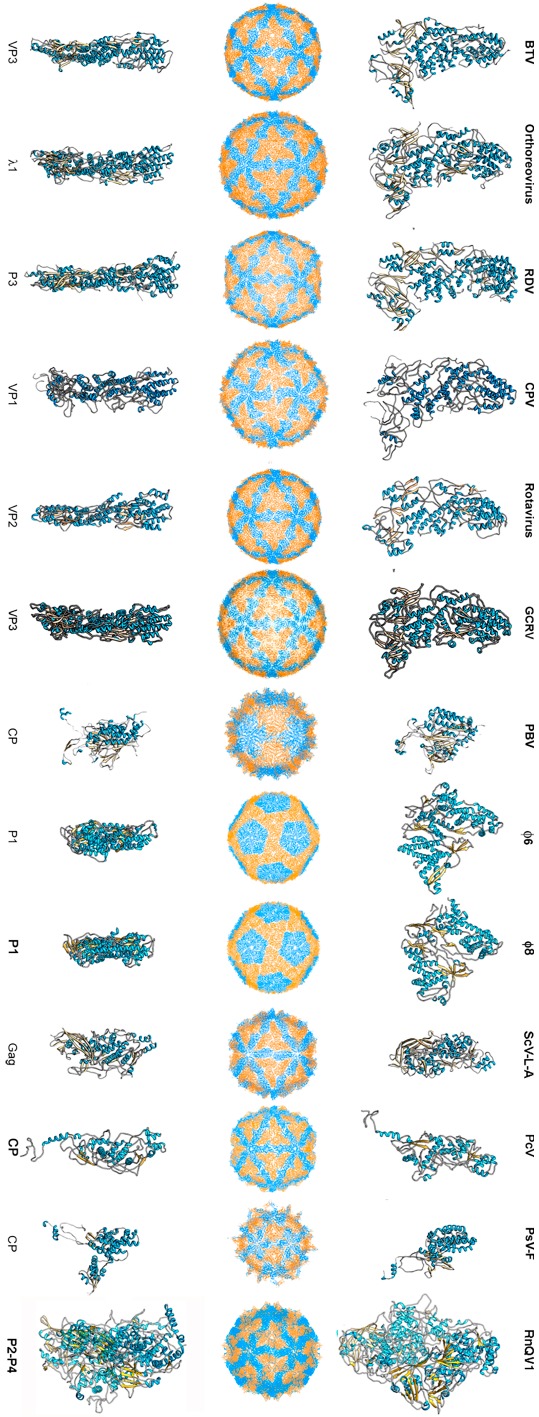
T = 1 capsid protein X-ray- and cryo-EM-based structures. Top row: T = 1 capsids of bluetongue virus (BTV), orthoreovirus, rice dwarf virus (RDV), cytoplasmic polyhedrosis virus (CPV), rotavirus, grass carp reovirus (GCRV), picobirnavirus (PBV), ϕ6 phage and ϕ8 phage, L-A virus of *Saccharomyces cerevisiae* (ScV-L-A), *Penicillium chrysogenum* virus (PcV), *Penicillium stoloniferum* virus F (PsV-F), and *Rosellinia necatrix* quadrivirus 1 (RnQV1), viewed along a two-fold axis of icosahedral symmetry (center row). BTV VP3 [2] (PDB accession number 2btv; 901 residues), λ1 [41] (1ej6; 1275 residues), P3 [42] (1uf2; 1019 residues), VP1 [19] (3cnf; 1333 residues), VP2 [43] (3kz4; 880 residues), GCRV VP3 [22] (3k1q; 1027 residues), PBV CP [23] (2vf1; 590 residues), ϕ6 P1 [44] (4btq; 769 residues) and ϕ8 P1 [45] (4btp; 792 residues), Gag [26] (1m1c; 680 residues), PcV CP domain A [46] (3j3i; 498 residues; complete CP 982 residues), PsV-F CP [29] (3es5; 420 residues) and RnQV1-W1118 P2 and P4 [36] (5nd1; 972 and 1005 residues, respectively), shown from top view. Bottom row: side views of the same structures (T = 1 shell exterior at right).

To date, 14 T = 1 capsid proteins have been resolved at the atomic level (Figure 1): VP3 of orbivirus bluetongue virus (BTV) [4], λ1 of reovirus (genus *Orthoreovirus*) [42], P3 of rice dwarf virus (RDV) [43], VP1 of cytoplasmic polyhedrosis virus (CPV) [20], VP2 of rotavirus [44], and VP3 of grass carp reovirus (GCRV) [23] (these last six viruses are all members of Reoviridae), CP of picobirnavirus (PBV) [24], ϕ6 P1 [45] and ϕ8 P1 [46] of the family *Cystoviridae*, Gag of the yeast virus ScV-L-A [27] (family Totiviridae), CP of *Penicillium chrysogenum* virus (PcV, family Chrysoviridae) [47], CP of *Penicillium stoloniferum* virus F (PsV-F, family Partitiviridae) [30], and the heterodimer P2–P4 of *Rosellinia necatrix* quadrivirus 1 (RnQV1, family Quadriviridae) [37]. The amino acid sequences of the above 14 CPs are quite different. All 14 proteins are, however, predominantly α-helical. The T = 1 CPs of *Reoviridae* members share an overall conformation [48], and those of PsV-F and PBV are based on similar folding. However, most CPs of mycoviruses have a tertiary structure that bears little resemblance to the reovirus structure. Rather, the 120-subunit capsids of fungal dsRNA viruses have a corrugated outer surface with protuberances rising above the continuous protein shell. Notably, the average thickness of a 120-subunit T = 1 CP is 15–30 Å in mammalian dsRNA viruses, but those of mycoviruses are thicker.

Unlike their bacteria- and higher eukaryote-infecting counterparts, most mycoviruses are transmitted by cytoplasmic interchange; they never leave the host, and indeed have no strategy for entering host cells [49]. Recent studies of fungal and protozoan dsRNA viruses identified functional and structural features unlike those recorded for members of the family *Reoviridae*, as well as evolutionary relationships among T = 1 capsid structural proteins. Whereas T = 1 capsids of reoviruses and cystoviruses share the same structural pattern, i.e., a 120-subunit capsid, most dsRNA mycoviruses exhibit high structural variability. ScV-L-A is built from 60 copies of a dimer of chemically identical subunits (as reo- and cystovirus), but the PcV T = 1 capsid is a variant of the 120-subunit capsid, as the CP has two motifs with the same fold, and the RnQV1 T = 1 capsid is composed of 60 dimers of two different proteins with a similar fold. In addition, the close relationship between the fungal dsRNA virus and its host probably place many constraints on the virus that it overcame by increasing CP complexity. In contrast to the plate-like protein found in reovirus and cystovirus T = 1 capsids, the 120-subunit capsid of fungal dsRNA viruses share a corrugated outer surface with domains rising above the continuous protein shell. In ScV-L-A virus, the CP has an extra domain with decapping activity, and the PcV and RnQV1 CP showed similar extra domains on the outer capsid surface with unknown functions. The present review focuses on the structure of dsRNA mycoviruses, and discusses how the lack of an extracellular phase has had unanticipated functional effects in their life cycles.

## 2. Structure of dsRNA Virus Capsids

### 2.1. Totiviruses

The L-A virus of the yeast *Saccharomyces cerevisiae* (ScV-L-A) is the type species of the genus *Totivirus* (family Totiviridae) [50,51]. The ScV-L-A genome is a 4.6 kb, single-segment dsRNA molecule that encodes a major capsid protein (Gag; 680 residues, 76 kDa) and viral polymerase (Pol; 868 residues, 94 kDa), as a Gag-Pol fusion protein generated by –1 ribosomal frameshifting [52,53]. Gag is bound covalently to the inside of the particle wall.

**Figure 2:**
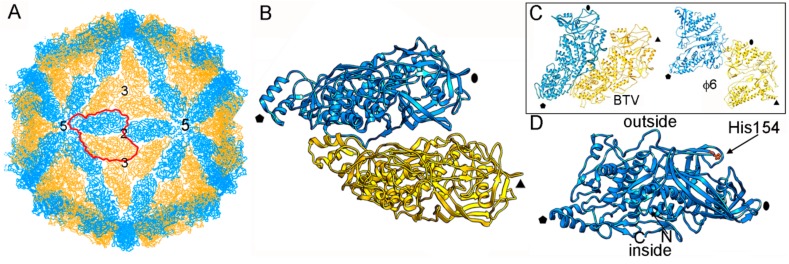
ScV-L-A T = 1 capsid protein; X-ray-based structure. (**A**) T = 1 capsid of ScV-L-A viewed along a two-fold axis of icosahedral symmetry, showing the Gag subunits A (blue) and B (yellow), with the boundaries of the asymmetric unit outlined in red. Numbers indicate icosahedral symmetry axes. (**B**) Atomic model of a Gag dimer (1m1c; 680 residues). Icosahedral symmetry five- (pentagon), three- (triangle), and two-fold (oval) axes are indicated in black. (**C**) T = 1 CP dimers of BTV (2btv) and ϕ6 (4btq). The structural unit is an asymmetric dimer in which subunits A and B are oriented in parallel with numerous side contacts. Symbols indicate icosahedral symmetry axes. (**D**) Side view of a Gag monomer (T = 1 shell exterior top). His154, the active site for decapping activity is indicated.

The structure of ScV-L-A was first examined by three-dimensional cryo-electron microscopy (3D cryo-EM) and later by X-ray crystallography (resolution 3.4 Å) [27]. Dark-field scanning transmission electron microscopy (STEM) was used to determine the virus stoichiometry [3,13]. The rough, icosahedral, ~400 nm diameter T = 1 lattice of ScV-L-A has 120 copies of Gag, of which one or two are fused to the Pol moiety [54] (Figure 2A). The protein shell is 56 Å thick. The structural unit is an asymmetric Gag dimer. Each Gag monomer can adopt one of two conformations, termed subunits A and B, with notable structural differences in specific surface regions and with entirely different bonding environments (non-equivalent contacts) (Figure 2B). These subunits are arranged in two sets of five: five A subunits directly surrounding the icosahedral five-fold axis, leaving an 18 Å diameter channel as a portal for the entry of nucleotide triphosphates and the exit of viral mRNA; and five B subunits intercalated between the A subunits, forming a decamer. This quaternary organization is similar to the 120-subunit T = 1 inner core of reoviruses [4,20,23,42,43,44] and cystoviruses [45,46], in which subunits A and B are arranged in nearly parallel positions (Figure 2C).

Gag functions as an enzyme and has a major role in the sophisticated interaction between ScV-L-A and the host cell. The Gag segment Gln139-Ser182, in which His154 is the active site, contributes to the rough outer surface of the capsid, and is responsible for the cellular RNA decapping activity that transfers the 7-methyl-GMP (m^7^GMP) cap from the 5′ end of the cellular mRNA to the 5′ end of the viral RNA [55,56] (Figure 2D). L-A counters a host exoribonuclease that targets uncapped RNAs (such as viral mRNA), allowing the latter to compete with host mRNA for use of the translation machinery.

The *Helminthosporium victoriae* virus 190S (HvV190S), a prototype of the genus *Victorivirus*, family Totiviridae, infects the filamentous fungus *H. victoriae*, and has a similar capsid organization to that of ScV-L-A [18,57]. The smooth HvV190S capsid (average thickness 35 Å) is composed of 120 CP monomers, with RdRp incorporated as a separate, non-fused protein synthesized by a stop/reinitiation mechanism [58,59]. The RdRp is either non-covalently associated with the underside of the capsid, as in reoviruses, free in the capsid interior, or non-covalently bound to the genome [18,57]. *Trichomonas vaginalis* virus 1 (TTV1), a totivirus of the genus *Trichomonasvirus* that infects a human-hosted protozoan, has its RdRp fused to the CP, as in ScV-L-A, but by −2 ribosomal frameshifting [29]. Notably, both the protozoan-infecting *Giardia lamblia* virus (GLV, genus *Giardiavirus*) [28] and the metazoan-infecting myonecrosis virus (IMNV, a tentative member of the family Totiviridae) [60,61] share the 120-subunit T = 1 capsid organization, but can be transmitted extracellularly.

### 2.2. Chrysoviruses

Chrysoviruses are isometric virions characterized by a multipartite genome [62]. *Penicillium chrysogenum* virus (PcV) is the prototype of the Chrysoviridae, a family of typically symptomless mycoviruses with a genome consisting of four monocistronic dsRNA segments (genome size 2.4–3.6 kbp). Each segment is encapsidated separately in a similar particle [63,64], i.e., chrysoviruses are multi-segmented and multi-particulate virions. dsRNA-1 (3.6 kbp) encodes the RdRp (1117 amino acid residues with a molecular mass of 128.5 kDa; one or two copies per virion), dsRNA-2 (3.2 kbp) encodes the CP (982 amino acid residues, 109 kDa), and dsRNA-3 and -4 (3 and 2.9 kbp) code for virion-associated proteins of unknown function (912 amino acid residues and 101 kDa, and 847 amino acid residues and 95 kDa, respectively).

**Figure 3:**
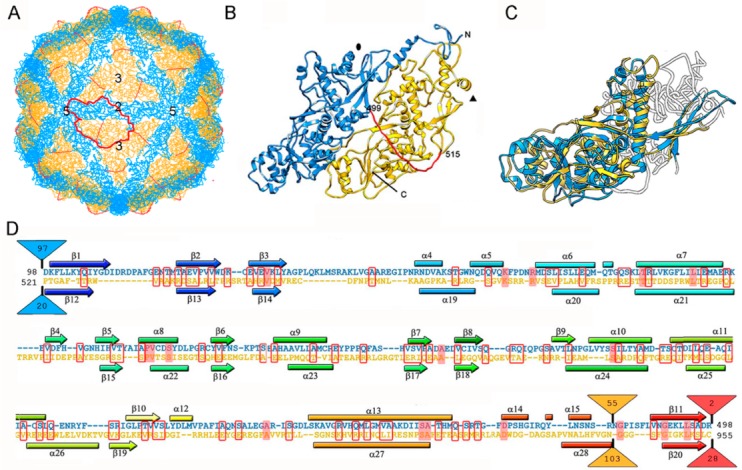
PcV T = 1 capsid protein; cryo-EM-based structure. (**A**) T = 1 capsid of PcV viewed along a two-fold axis of icosahedral symmetry, showing the N-terminal domain A (1–498, blue), the linker segment (499–515, red), and the C-terminal domain B (516–982, yellow), with the boundaries of an asymmetric unit outlined in red. Numbers indicate icosahedral symmetry axes. (**B**) Top view of the atomic of the PcV CP (3j3i; 982 residues). Symbols indicate icosahedral symmetry axes. (**C**) PcV capsid protein is a structural duplication. Superimposed A and B domains (white segments indicate non-superimposed regions for both domains). (**D**) Sequence alignment of domains A (blue) and B (yellow) resulting from Dali structural alignment. α-helices (rectangles) and β-strands (arrows) are rainbow-colored from blue (N terminus) to red (C terminus) for each domain. Triangles represent non-aligned segments (sizes indicated): the orange triangle indicates the single “hotspot” on the outer capsid surface. Strictly conserved residues are on a red background and partially conserved residues are in a red rectangle.

So far, the 3D structures of the capsids of two chrysoviruses have been determined by cryo-EM analysis, that of PcV at atomic resolution [47], and that of *Cryphonectria nitschkei* chrysovirus virus 1 (CnCV1) at subnanometer resolution [35]. Analytical ultracentrifugation analysis has shown that PcV and CnCV1 virions are exceptions to the most-extended tendency among dsRNA viruses—a T = 1 core with 60 equivalent dimers—since they have an authentic T = 1 capsid formed by 60 copies of a single monomer [33,34]. The capsid diameter is 400 Å and the protein shell is 48 Å thick (Figure 3A). Similar to ScV-L-A, the outer capsid surface of PcV is relatively uneven with 12 outwardly protruding pentons, each containing five copies of the CP; this contrasts with the smooth outer surface of reoviruses, in which the CP has a plate-like structure. The 982-residue CP of PcV is formed by duplication of an α-helical domain; this is indicative of gene duplication despite negligible sequence similarity between the two roughly parallel α-helical domains (Figure 3B). The N-terminal A domain (residues 1–498) and the C-terminal B domain (residues 516–982) are connected by a 16-residue linker (Ala499-Ile515), accessible from the capsid outer surface. These domains are arranged in two sets of five: five A domains directly surround the icosahedral fivefold axis and five B domains intercalated between them, forming a pseudodecamer. This organization is clearly reminiscent of the 120-subunit T = 1 lattice of totivirus and megabirnavirus (as well as reovirus and cystovirus) capsids, in which the two asymmetrical dimer components are arranged in near-parallel fashion. The structural details of the PcV capsid reinforce the idea that a T = 1 layer with a dimer as the asymmetric unit provides an optimal framework for managing dsRNA metabolism.

Superimposition of the PcV A and B αhelical domains identifies a single “hotspot” on the outer capsid surface where variation is introduced by insertion of 50–100 residue segments (Figure 3C,D). A preferential insertion site would allow the acquisition of new functions while preserving basic CP folding. It is plausible that, in addition to its structural role, chrysovirus CP also has enzymatic activity.

### 2.3. Partitiviruses

Members of the family Partitiviridae have bisegmented, 1.4–2.4 kbp-long genomes. Each segment is encapsidated separately in a similar virus particle. dsRNA1 encodes RdRp (one copy per virion), whereas dsRNA2 encodes the CP. The partitiviruses that infect fungi are grouped into three genera: alpha-, beta-, and gamma-partitiviruses [65,66]. Alpha- and beta-partitiviruses infect plants and filamentous fungi, whereas gamma-partitiviruses infect only the latter. In general, partitivirus infections are largely symptomless.

Four fungal partitivirus structures have been resolved by 3D cryo-EM, including those of the gamma-partitiviruses *Penicillium stoloniferum* virus S (PsV-S) [67,68] and *Penicillium stoloniferum* virus F (PsV-F) (by X-ray crystallography at 3.3 Å resolution) [30], and of the beta-partitiviruses *Fusarium poae* virus 1 (FpV1) [31] and *Sclerotinia sclerotiorum* partitivirus 1 (SsPV1) [69].

**Figure 4:**
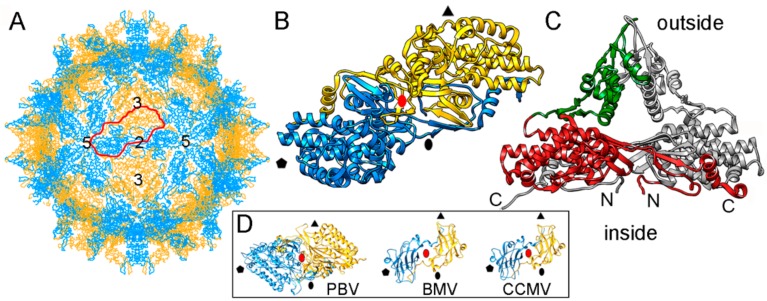
PsV-F T = 1 capsid protein X-ray-based structure. (**A**) T = 1 capsid of PsV-F viewed along a two-fold axis of icosahedral symmetry, showing the CP subunits A (blue) and B (yellow), with the boundaries of an asymmetric unit (a quasi-symmetric A-B dimer) outlined in red. Numbers indicate icosahedral symmetry axes. (**B**) Top view of the atomic model of a PsV-F CP dimer (3es5; 420 residues). Symbols indicate icosahedral symmetry axes; red oval indicates a local two-fold symmetry axis. (**C**) Side view of a PsV-F CP dimer. The arch (green) and shell domains (red) are indicated. For clarity, the second subunit is shown in grey. (**D**) T = 1 CP dimers of PBV (2vf1), BMV, brome mosaic virus (1js9), and cowpea chlorotic mottle virus (CCMV; 1cwp). Dimers are related by a local quasi-two-fold symmetry axis (red oval), and show molecular swapping. BMV and CCMV are plant ssRNA viruses with T = 3 capsids, but their CP (with a β-barrel domain) can assemble into 120-subunit capsids that show a quaternary organization similar to that of PsV-F and PBV.

The single-layered, 120-subunit capsids of these viruses are 35–42 nm in diameter and distinct in that they have “arch-like” surface features that protrude above the continuous capsid shell (Figure 4A). These T = 1 capsids have a different quaternary organization, their CP dimer having almost perfect local two-fold symmetry (Figure 4B). The quasi-symmetric CP dimer is stabilized by domain swapping within the shell region of the A and B subunits, as well as by intradimeric interactions between equivalent protruding arch domains on the particle surface (Figure 4C). A similar organization has been found in a picobirnavirus [24]—a bisegmented dsRNA virus that infects humans and other vertebrates. This might represent convergent evolution. Brome mosaic virus (BMV) and cowpea chlorotic mottle virus (CCMV), two plant positive-sense (+) ssRNA viruses with a T = 3 capsid, have a CP (with a β-barrel domain) that assembles into a 120-subunit capsid with a quaternary organization similar to that of PsV-F and PBV [70,71] (Figure 4D).

Based on their capsid organization, partiti- and picobirnaviruses appear to be assembled from dimers of CP dimers (i.e., tetramers). In contrast, the proposed assembly pathway for the 120-subunit capsids of Totiviridae and Reoviridae members is based on pentamers of CP dimers (i.e., decamers). Notably, the capsid protein P1 of bacteriophage ϕ8 (a cystovirus) appears as a soluble tetramer in an in vitro assembly system [72].

### 2.4. Quadriviruses

*Rosellinia necatrix* quadrivirus 1 (RnQV1) is the type species of the genus *Quadrivirus* in the family Quadriviridae [73,74]. The filamentous ascomycete *Rosellinia necatrix*, a pathogen of many plants, can be infected by dsRNA viruses belonging to at least six families [75,76]. RnQV1 is associated with latent infections (i.e., it causes no apparent slowing of host growth), and has a multipartite genome consisting of four monocistronic dsRNA segments (as in chrysoviruses) with genome sizes ranging from 3.7 to 4.9 kbp. DsRNA-1 (4.9 kbp) codes for a protein of unknown function (1602 amino acid residues), dsRNA-2 (4.3 kbp) encodes the P2 CP (1356 amino acids), dsRNA-3 (4 kbp) codes for RdRp (1117 amino acids), and dsRNA-4 (3,7 kbp) codes for the P4 CP (1061 amino acids).

**Figure 5:**
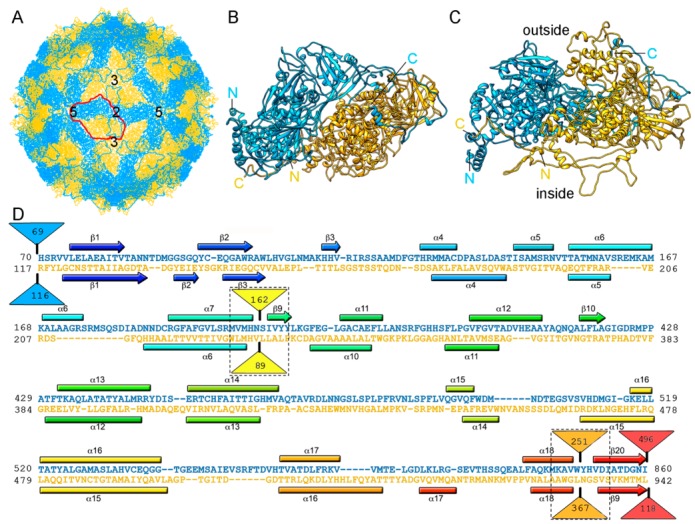
RnQV1 T = 1 capsid cryo-EM-based structure. (**A**) T = 1 capsid of RnQV1 viewed along a two-fold axis of icosahedral symmetry, showing P2 (blue) and P4 (yellow). Boundaries for an asymmetric unit are outlined in red. Numbers indicate icosahedral symmetry axes. (**B**) Top and (**C**) side views of the atomic models of P2 (blue; 972 residues) and P4 (yellow; 1005 residues) (5nd1). The last visible P2 C-terminal residue is located on a P4 surface crevice. (**D**) Sequence alignment of P2 (blue) and P4 (yellow) resulting from Dali structural alignment. α-helices (rectangles) and β-strands (arrows) are rainbow-colored from blue (N terminus) to red (C terminus) for each protein. Dashed rectangles indicate favorable insertion sites, triangles represent non-aligned segments (sizes indicated).

RnQV1 virus strains W1075 and W1118, isolated from different locations in Japan, have been analyzed by 3D cryo-EM and analytical ultracentrifugation [36]. Their P2 and P4 proteins co-assemble into isometric virus particles ~45 nm in diameter, which each package either one or two of the four genome segments. Whereas most dsRNA virus capsids are based on dimers of a single protein, RnQV1 has a single-shelled T = 1 capsid formed by 60 P2 and P4 protein heterodimers (Figure 5A). Whereas P2 and P4 of RnQV1 strain W1118 remain nearly intact, in strain W1075, both proteins are cleaved into discrete polypeptides, apparently without altering capsid structural integrity. The atomic structure of the RnQV1 W1118 capsid at 3.7 Å resolution shows that P2–P4 heterodimers are organized into a quaternary structure similar to that of the homodimers of reoviruses, chrysoviruses, and totiviruses [37] (Figure 5B,C). Although the RnQV1 capsid, and that of PcV, is an exception to the rule that all dsRNA viruses have a T = 1 capsid with a CP homodimer as the asymmetric unit, it follows the architectural principle that a 120-subunit capsid is a conserved assembly that supports dsRNA replication and organization.

Despite their low sequence similarity, the superimposition of P2 and P4 revealed their having a common α-helical domain (Figure 5D). As described for the PcV capsid, P2 and P4 have also acquired new functions through the insertion of complex domains at preferential insertion sites on the capsid outer surface. These are also probably related to enzyme activity. The P2 insertion has a fold similar to that of gelsolin and profilin, two actin-binding proteins with a function in cytoskeleton metabolism; whereas the P4 insertion suggests a protease activity involved in cleavage of the P2 383-residue C-terminal region (absent in the mature viral particle). This P2 C-terminal segment might represent an external scaffolding domain [37].

## 3. Evolutionary Relationships Based on Structural Comparisons

Structural comparisons of CPs have been used to establish relatedness when sequence conservation is limited [77,78,79], and have detected relationships among viruses that infect organisms that, in evolutionary terms, are widely separated [78,80,81,82]. Icosahedral viruses are grouped into four lineages [80]: the dsDNA viruses with an upright double β-barrel CP (the prototypes are phage PRD1 and adenoviruses), the head-tailed phages and herpesviruses that share the Hong Kong 97 (HK97)-like CP fold (also termed the Johnson fold), the picornavirus-like superfamily with a single β-barrel as the CP fold, and the dsRNA or bluetongue virus (BTV)-like viruses. The PRD1- and HK97-like lineages include archaea-, bacteria-, and eukaryote-infecting viruses, suggesting that their last common ancestral hosts were infected by the progenitors of the current viral lineages before the host organisms diverged [82,83,84]. Although many viruses are not included in these four lineages, the number of folds that satisfy the assembly constraints for a viable viral shell is thought to be limited.

**Figure 6:**
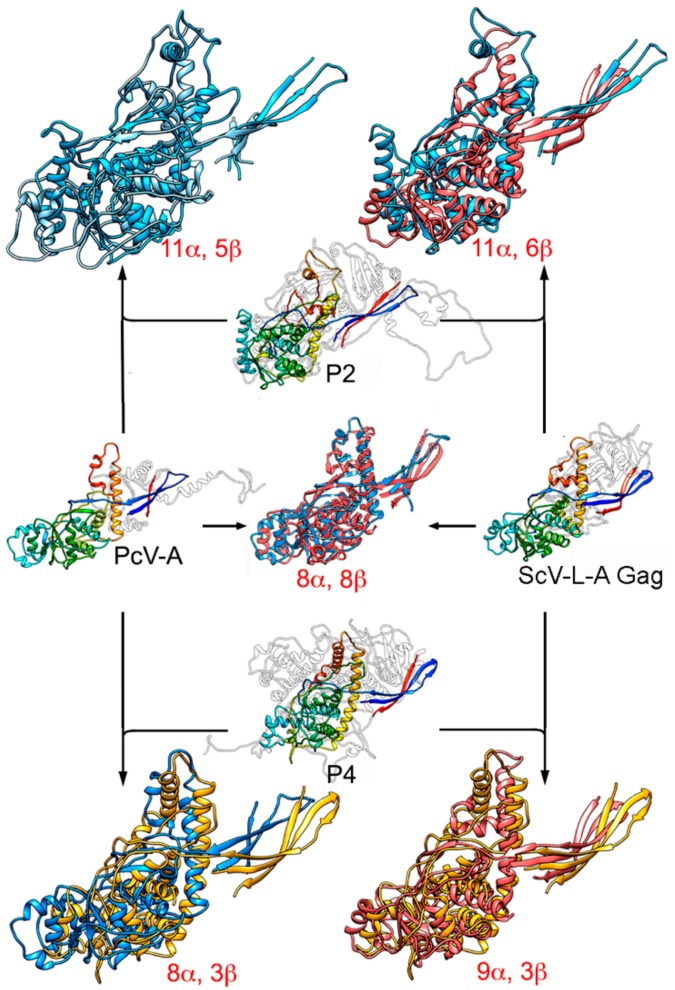
Structural homology of mycovirus T = 1 CP. The PcV CP A domain (PcV-A, left, center) was structurally aligned with ScV-L-A Gag (right, center), and P2 (top, center) and P4 (bottom, center) with PcV-A and ScV-L-A Gag. Center rainbow-colored structures indicate conserved secondary structure elements within the dsRNA viruses. PcV-A is aligned with ScV-L-A Gag (blue and pink, center). P2 is aligned with PcV-A (blue and light blue, top left) and with ScV-L-A Gag (blue and pink, top right). P4 is aligned with PcV-A (yellow and blue, bottom left) and P4 with ScV-L-AL-A Gag (yellow and pink). Total numbers of secondary structural elements with close relative spatial locations are indicated.

The similarity of the A and B α-helical domains of PcV CP, which have many well-matching secondary structural elements, indicate a common fold in both domains [47]. Gene duplication (or joined folds) has been a recurrent evolutionary event in other viral lineages, for example, involving the trimeric capsomeres of adenoviruses [85], *Paramecium bursaria* chlorella virus 1 (PBCV1) [86], and bacteriophage PRD1 [87], and the large subunit of comoviruses ([+] ssRNA viruses that infect plants) [88]. The conserved ~350 residue-long PcV fold is also preserved in the Gag of ScV-L-A [33,47] (Figure 6). This basic α-helical domain shares many secondary structural elements with L-A Gag, in particular those regions involved in interactions at the five-, three-, and two-fold symmetry axes. The preserved fold in Gag has three peptide insertion sites facing the outer capsid surface, one of which colocalizes with the single-insertion hotspots of the PcV CP domains. This colocalization suggests that these preferential insertion sites are ancient, and provide a means for the acquisition of new functions without altering the structural and functional motifs of the dsRNA virus CP.

P2 and P4 of RnQV1 also have a common fold some 300 residues long, with two preferential insertion sites on the outer surface [37]. Both coincide with the ScV-L-A Gag insertion sites, and one with the single-insertion site of the PcV A and B α-helical CP domains. Notably, the conserved folds of PcV and ScV-L-A CP are similar to the common fold of P2 and P4, indicating that this fold may have evolved from a common ancestral domain of the dsRNA virus lineage (Figure 6).

Despite their size and overall shape differences, the preserved ~300-residue PcV domains can be compared with the 1000–1300-residue reovirus T = 1 CP through the use of robust structural alignment methods for highly diverged CP structures [46,89]. There are discernible similarities in the arrangement of the secondary structural elements that place ϕ8 CP as an intermediate between reovirus CP and mycovirus CP [46], i.e., at the furthest distance within the structure-based phylogenetic tree. The preserved α-helical domain of mycoviruses is broken by much longer insertions in reovirus CP, resulting in basic structural motifs or subdomains.

Duplication of an ancestral gene for a CP with the BTV-like fold might have resulted in two separate (as in quadriviruses) or covalently joined folds (as in chrysoviruses). This event could direct the assembly of a T = 1 capsid with 120 subunits or domains with a dimer as the asymmetric unit—a necessary arrangement for dsRNA replication/transcription. Separate and joined folds are found in the CP of other virus families, such as picornaviruses [79] and comoviruses [88], respectively. Once the 120-subunit capsid was well-established, later divergent evolutionary events would have introduced additional changes in each copy, or even the complete removal of one of them, producing a CP that assembles as a dimer of unfused identical monomers. Alternatively, the ancestral CP could have initially acquired dimer assembly ability, followed by gene duplication.

The CP of many-tailed dsDNA phages with the HK97-like fold has additional domains with specific functions related to capsomere and/or capsid stability (reviewed in Suhanovsky, M.M. et al. [90]). Human cytomegalovirus (HCMV), a herpesvirus, has a 1370-residue CP folded into seven domains [91], with the Johnson fold or floor domain in the shell, and a six-domain protruding tower. The Johnson fold has a five-stranded β-core that acts as the organizational hub of the CP; the additional domains in the Johnson fold are considered modular insertions into the peripheral loops [91]. In this context, tailed dsDNA phages and herpesviruses share some similarities with dsRNA mycoviruses. Conserved α-helices and/or the β-sheet structure preserved in the dsRNA virus basic fold might form a similar functional center for domain insertion.

## 4. RdRp and dsRNA Organization within Mycovirus Capsids

Reovirus T = 1 cores have 10–12 RdRp complexes per virion, around which the dsRNA is densely coiled [92,93]. RdRp complexes are non-covalently anchored to the capsid inner surface near the icosahedral 5-fold axes [19,94,95], as presumably they are in mycoreoviruses. In addition to RdRp molecules, reovirus replicase complexes include a few minor core proteins with ATPase- and/or RNA-binding abilities. For members of the Toti-, Chryso- Partiti-, and Quadriviridae families, the RdRp molecules are incorporated into one or two copies per virion, and show more variability than reovirus. For chryso-, partiti-, and quadriviruses, the RdRp is expressed as a physically separate protein from a discrete genome segment, and is incorporated into virions via non-covalent interactions with the capsid and/or genome. The same is true for victoriviruses (genus *Victorivirus*, family Totiviridae), such as HvV190S, except that the RdRp is expressed from the single genome segment of those viruses via a coupled termination-reinitiation mechanism [58,59,96]. For totiviruses such as ScV-L-A (genus *Totivirus*), in contrast, the RdRp is expressed as a C-terminal fusion product with the CP (i.e., as a Gag-Pol). As a result, in ScV-L-A, the one or two RdRp domains per virion are covalently tethered to the capsid via the fused CP domain, which occupy one or two subunit positions in the capsid.

The anchoring of RdRp at the five-fold axes on the reovirus capsid inner surface seems likely to occur in toti-, chryso-, partiti-, and quadriviruses too, with important consequences for the channeling of freshly synthesized transcripts into an exit pore.

**Figure 7:**
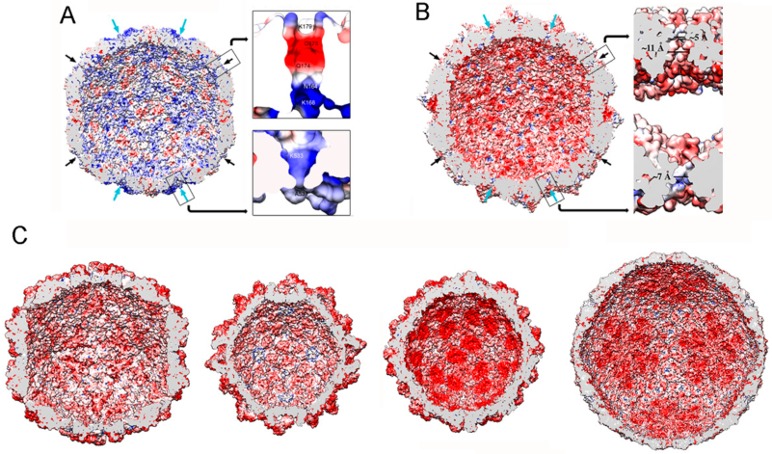
dsRNA virus T = 1 capsid inner surfaces with electrostatic potentials. (**A**) PcV and (**B**) RnQV1 capsid inner surfaces viewed along a two-fold axis of icosahedral symmetry. The inner surface charge representations of these capsids show the distribution of negative (red) and positive (blue) charges. Note the numerous electropositive areas in PcV. Arrows indicate the capsid pores at the five-fold (black) and three-fold (blue) axes. Boxes: magnified views of the five- (top) and three-fold (bottom) pores showing charge distribution on the channel walls. (**C**) T = 1 capsids of ScV-L-A, PsV-Fe, rabbit picobirnavirus, and rotavirus (from left to right), viewed along a two-fold axis of icosahedral symmetry. dsRNA and packaged proteins (such as RNA polymerases) were removed computationally.

The mycovirus T = 1 capsid wall is perforated by many pores and channels, but none is large enough to pass an A-form 23-Å-diameter duplex (Figure 7A,B). Whereas the largest pores (15–20 Å diameter and usually located near the five-fold axis) would allow the passage of nascent mRNA into the host cytoplasm, the smallest holes (5–10 Å in diameter and usually located at the three-fold axis) could be used for nucleotide substrate or pyrophosphate byproduct diffusion. In non-transcribing T = 1 capsids, the pores are very narrow, but the N- or C-termini or the side chains of residues that face the channel wall might adopt alternative conformations to allow the exit of viral transcripts.

With the exception of totiviruses, which have a single genomic segment, many fungal dsRNA viruses, including chryso-, partiti-, and quadriviruses, have multisegmented dsRNA genomes. In addition, the multisegmented viruses appear to be multiparticulate, i.e., segments are encapsidated separately [97]. Fungal dsRNA viruses have spacious capsids in comparison with the inner cores of complex eukaryotic dsRNA viruses (Table 1). Whereas reoviruses have 9–12 genome dsRNA segments packed into liquid crystalline arrays at high density (~40 bp/100 nm^3^, a spacing between dsRNA strands of 25–30 Å) [6,98,99,100], fungal virus capsids (including ScV-L-A, PcV, PsV-F, and RnQV1) contain a single loosely packed dsRNA molecule (~20 bp/100 nm^3^, an interstrand spacing of ~40–45 Å) [34,49,63]. In reoviruses, individual genome segments must be transported through the active sites of the RdRp complexes at the five-fold axes, and template motion could be a limiting factor. ScV-L-A is a simplified version of these viruses, with a single-segment genome. The looser packing of the dsRNA would probably improve template motion in the more spacious transcriptional and replicative active particles, minimizing electrostatic repulsion between dsRNA strands.

Most mycovirus T = 1 capsids are negatively charged on their inner surface, a feature common to many such capsids of dsRNA viruses [37]. This might facilitate the movement of template and/or product RNA molecules by repulsion, maintaining the RNA layer at ~25 Å from the capsid surface (Figure 7B,C). The PcV capsid is an exception. It has positively charged regions on the inner surface (Figure 7A) and has numerous interactions with the underlying genome, which is ordered in the outermost RNA layer [33]. As a result, there is almost no space between the latter layer and the inner capsid surface. These contacts have been defined at the atomic level in PcV and PsV-F virions [30,47]. The lower density of the central region and the associated slight increase in dsRNA mobility might be necessary for maximum RdRp activity in the context of a non-fused RdRp complex.

Comparative analysis of dsRNA packing densities in dsRNA virions have revealed two major tendencies among T = 1 capsids of dsRNA viruses: (1) those with 9–12 dsRNA segments densely packaged within the same particle and containing 9–12 RdRp complexes, as seen in reoviruses, and (2) those with a single-genomic dsRNA segment with less internal order and one or two copies of the RdRp complex per particle, as seen in mycoviruses.

## 5. Concluding Remarks and Future Perspectives

Structural studies of a limited number of fungal viruses have revealed them to conform to the basic concepts of dsRNA viruses, but also to have unexpected features that have contributed to a better understanding of their structure, function, and evolution. dsRNA mycovirus capsids, exemplified by ScV-L-A, PcV, PsV-F, and RnQV1, show structural variations of the same framework optimized for RNA metabolism; they possess 60 asymmetric or symmetric dimers of a single protein (ScV-L-A and PsV-F, respectively), dimers of similar domains (PcV), or dimers of two different proteins (RnQV1). Since mycoviruses are transmitted by cytoplasmic interchange and commonly confined to their hosts, their capsids incorporate polypeptides and domains on their outer surfaces for the acquisition of new functions without altering the structure and function of the CP. Such acquisitions would eventually lead to optimal viral-host interactions.

Despite recent advances in understanding the structure of dsRNA mycoviruses, many aspects of several fungal (and protozoan) viruses remain unknown. Recent work has identified a positive-sense ssRNA virus—the yado-kari virus 1 (YkV1)—that hijacks the CP of a dsRNA virus that resembles totivirus—the yado-nushi virus 1 (YnV1) [101,102]. There are several papers reporting yadokari-like viruses with sequence similarity to YkV1 [102,103,104,105], but their possible mutualism with potential partners has yet to be elucidated. Another notable example includes Aspergillus fumigatus tetramycovirus 1 (AfuTmV1) [106], Colletotrichum camelliae filamentous virus 1 (CcFV-1) [107], and related viruses. Despite similarity in genome organization and sequence, these viruses seemingly utilize different genome packaging strategies; namely, the genomic dsRNAs are associated with a virally encoded protein in a colloidal form (AfuTmV1) or packaged in filamentous particles (CcFV-1), for which infectivity as purified dsRNA has also been demonstrated. Future structural studies should focus on the asymmetric substructures and components of their capsids [108,109]—such as their RdRp (isolated or packaged inside virions)—and their packaged dsRNA genome.

## Figures and Tables

**Table 1:** Genome packaging densities in double-stranded ribonucleic acid (dsRNA) viruses.

Virus Family	dsRNA Features			Capsid Features		
	Nº Segments	Size (kbp)	MW ^c^ (MDa)	CP (residue)	Φ ^d^/ir ^e^ (nm)	dsRNA Density (bp/100 nm^3^) ^f^
**HSV ^a^**	1	~152	103.7	1374	~130/43	46
***Reoviridae***						
*Orthoreovirus*	10	~23.5	16	1275	~60/24.5	38
*Rotavirus*	11	~18.5	12.6	880	~52/23.5	34
*Orbivirus*, BTV	10	~19.2	13.1	901	~52/22	43
*Aquareovirus*, GCRV	11	~23.6	16	1027	~60/23	46
*Phytoreovirus*, RDV	12	~25.7	17.5	1019	~57/26	35
*Cypovirus*, CPV	10	~31.4	21.4	1333	~58/24	54
*Mycoreovirus*, MyRV1	11	23.4	16			
***Picobirnaviridae***	2	~4.2	2.9	590	~35/14	
***Cystoviridae***, phage ϕ6	3	~13.4	9.1	769	~50/20	40
***Totiviridae***, ScV-L-A	1	~4.6	3.1	680	~43/17	22
***Partitiviridae***, PsV-S	1 (2) ^b^	~1.7 (3.3)	1.2 (2.2)	420	~35/12	23
***Chrysoviridae***, PcV	1 (4) ^b^	~3.2 (12.6)	2.2 (8.6)	109	~40/16	19
***Megabirnaviridae***, RnMBV1	1 (2) ^b^	~8.1 (16.2)	5.5 (11)	135	~52/19	28
***Quadriviridae***, RnQV1	1–2 (4)	~4.3 (17.1)	2.9 (11.7)	1356 + 1061	~47/16	25 (50) ^g^

^a^ Herpes simplex virus, a dsDNA virus with liquid-crystalline packing of the encapsidated, B-form dsDNA. ^b^ For PsV-S, PcV, and RnMBV1 dsRNA, the genome is formed by two, four, or two dsRNA molecules, respectively, but a mean value was calculated for one dsRNA molecule/particle in each column. ^c^ Molecular weights (MW) were calculated assuming a mass of 682 Da/bp. HSV dsDNA is assumed to have a B-form. ^d^ Outer diameter. ^e^ Inner radius. ^f^ Densities when volume of a perfect sphere is assumed and any other internal components are ignored. ^g^ 25 if there is one dsRNA molecule/particle; 50 if there are two dsRNA molecules/particle.
